# Novel approach to investigate the association between type 2 diabetes risk and dietary fats in a dietary pattern context: a scoping review

**DOI:** 10.3389/fnut.2023.1071855

**Published:** 2023-06-01

**Authors:** Barbara Brayner, Gunveen Kaur, Michelle A. Keske, Laura E. Marchese, Katherine M. Livingstone

**Affiliations:** School of Exercise and Nutrition Sciences, Institute for Physical Activity and Nutrition, Deakin University, Geelong, VIC, Australia

**Keywords:** dietary patterns, dietary fats, type 2 diabetes, reduced rank regression (RRR), review

## Abstract

The effect of dietary fat on type 2 diabetes (T2D) risk is unclear. *A posteriori* dietary pattern methods have been increasingly used to investigate how dietary fats impact T2D risk. However, the diverse nutrients, foods and dietary patterns reported in these studies requires examination to better understand the role of dietary fats. This scoping review aimed to systematically search and synthesize the literature regarding the association between dietary patterns characterized by dietary fats and T2D risk using reduced rank regression. Medline and Embase were searched for cross-sectional, cohort or case-control studies published in English. Of the included studies (*n* = 8), five high-fat dietary patterns, mostly high in SFA, were associated with higher T2D risk or fasting glucose, insulin and Homeostasis Model Assessment (HOMA) levels. These were mostly low-fiber (*n* = 5) and high energy-density (*n* = 3) dietary patterns characterized by low fruit and vegetables intake, reduced fat dairy products and higher processed meats and butter intake. Findings from this review suggest that *a posterior*i dietary patterns high in SFA that increase T2D risk are often accompanied by lower fruits, vegetables and other fiber-rich foods intake. Therefore, healthy dietary fats consumption for T2D prevention should be encouraged as part of a healthful dietary pattern.

## Introduction

Type 2 diabetes (T2D) is a chronic condition characterized by elevated glucose levels, or hyperglycaemia (1). If left untreated, chronic hyperglycaemia can lead to an increased risk of cardiometabolic complications and early death (1). In the past 30 years, the prevalence of T2D has increased from approximately 108 million to 422 million adults worldwide (2). The projections estimate that by 2045, 700 million individuals will develop diabetes (3). Therefore, understanding how T2D progresses is critical for developing preventative methods to lower its incidence worldwide.

An unhealthy diet is considered one of the key risk factors for the onset of T2D (4). Whilst the benefits of managing some dietary risk factors, such as carbohydrate intake, have been well researched (5) the role of dietary fats is less clear and this is likely due to the varying sources of fat in our diet (6). Higher polyunsaturated fatty acids (PUFA) intake has been linked to lower fasting blood glucose levels (7) and reduced T2D incidence (8, 9), whilst higher saturated fatty acid (SFA) intake has been linked to the development of insulin resistance (10). However, SFA intake may also differentially affect the risk of developing T2D, depending on its food source; SFA from meat may increase risk (11, 12) whereas SFA from dairy may decrease risk of T2D (13). Moreover, some studies also report no association between dietary fat type and risk of T2D (6, 14). The majority of studies to date have focused on a single nutrient approach (10, 14–16). However, as foods and nutrients are not eaten in isolation, understanding of the role of dietary fat within the context a dietary pattern is warranted.

There are three main delineations of dietary pattern methodologies: *a priori*, empirical and *a posteriori* (16, 17). The *a priori*, or hypothesis-oriented approach, derives dietary patterns using a pre-defined criterion (16) whilst empirical methods use a data-driven approach to create dietary patterns according to the combinations of foods consumed in a specific population (16). Extensive research has been conducted to investigate the association between *a priori* and empirical dietary patterns and T2D (18, 19). However, the use of *a posteriori* dietary pattern methods to understand T2D is only beginning to be explored.

*A posteriori* methods, such as reduced rank regression, combine both hypothesis-oriented and data driven approaches to create patterns of food intake according to pre-defined response variables (16, 20, 21). The response variables selected for use in reduced rank regression are known to be on the causal pathway between diet and the health outcome of interest, which can include biomarkers or nutrient intake. While increasing research has used reduced rank regression to investigate the association between diet and T2D using biomarkers as response variables (22–25) few studies have used nutrient intakes as response variables (16, 26–28). An understanding of how dietary fats, within the context of overall dietary patterns, impact on T2D risk is important for advancing T2D research beyond a single nutrient-focus. Therefore, the aim of this scoping review was to systematically search and synthesize the literature regarding the association between dietary fats and risk of T2D while using reduced rank regression.

## Methods

### Eligibility criteria

This scoping review included publications from human observational studies (cross-sectional and cohort studies) from inception to November 2021. To be eligible for inclusion, studies were required to include information on: (i) *a posteriori* dietary patterns that used dietary fat [saturated fat (SFA), monounsaturated fat (MUFA), polyunsaturated fat (PUFA), total fat, unsaturated fat, omega-3, omega-6, eicosapentaenoic acid (EPA), docosapentaenoic acid (DPA), docosahexaenoic acid (DHA), arachidonic acid (AA)] as at least one of the response variables regardless of the other response variables (could be nutrient or biomarker); and (ii) T2D risk, insulin resistance, homeostasis model assessment of insulin resistance (HOMA-IR), fasting insulin, fasting glucose, glycated hemoglobin (HbA1c), oral glucose tolerance test (OGTT) or gestational diabetes as an outcome. As a preliminary search identified few studies that investigated T2D as an outcome, the authors have included related outcomes to provide a more comprehensive overview of the research question. All population groups were included. Non-English publications were excluded. This scoping review was undertaken in accordance with Preferred Reporting Items for Systematic Reviews and Meta-analysis—Extension for Scoping Reviews (PRISMA-ScR) (29).

### Search strategy

#### Information sources

Two electronic databases (Medline and Embase) were searched in November 2021. An updated search was run in July 2022.

#### Literature search

The search strategy was developed and piloted in consultation with a librarian. It involved combining two search themes using the Boolean operator “and”, while only searching titles and abstract. The first theme was (“reduced rank regression” or “rrr”) and the second theme was (“type 2 diabetes” or “T2D” or “T2DM” or “type 2 diabetes mellitus” or “TIIDM” or “insulin resistance” or “insulin resistant” or “IR” or “impaired glucose tolerance” or “pre-diabetes” or “glucose intolerance” or “impaired fasting glucose” or “gestational diabetes” or “GDM”). To identify all possible studies that used reduced rank regression, “fat” and “dietary fats” was not used as a search term. Instead, these terms were included during the screening of titles and abstracts by two independent reviewers.

#### Selection of sources of evidence

The search results were exported to Covidence. Initial screening of articles titles and abstracts was performed by two independent reviewers (BB and LM) according to the inclusion and exclusion criteria. If both reviewers agreed on the suitability of the article, it was moved to full text screening and again reviewed independently by two reviewers. If there was any disagreement, a third reviewer (KML) was consulted. Duplicates were removed via the in-built function in Covidence.

#### Data extraction

Data extraction was performed by using a pre-piloted Excel template. The following information was extracted: (i) study design, sample size; (ii) dietary assessment method; (iii) dietary patterns: food groups (predictors) and nutrient intakes (response variables); (iv) outcomes; (v) adjustments made in the analysis; (vi) main results. In studies investigating multiple outcomes, only the outcomes listed in the eligibility criteria were summarized. Where response variables included a combination of nutrient intakes and other measures, these were also extracted.

#### Synthesis of results

A narrative approach was used to summarize the main findings of included studies. Results were presented by grouping included studies based on their outcome to better describe the level of evidence for T2D and related outcomes and highlight any gaps in knowledge.

## Results

**Figure 1:**
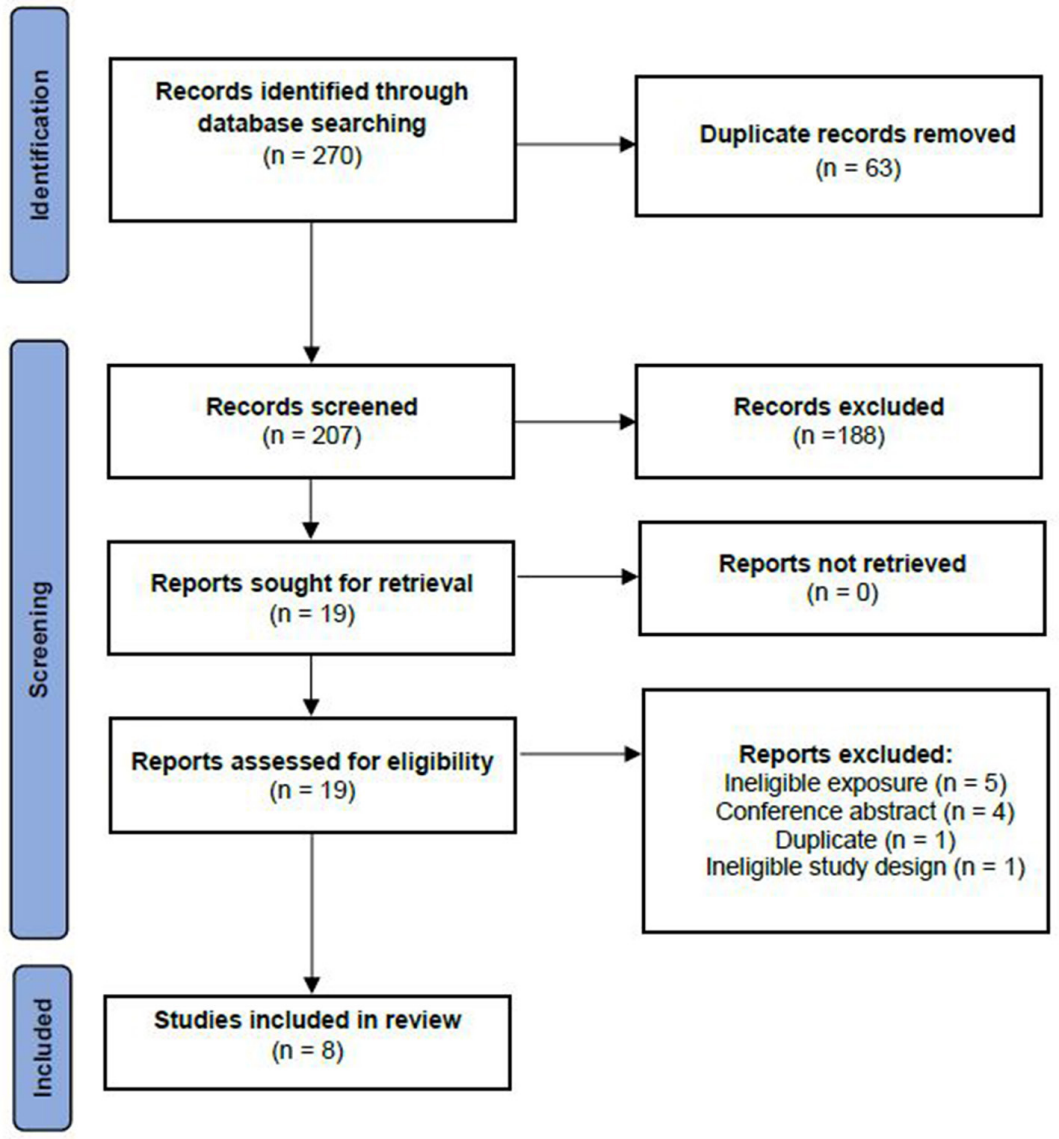
Study selection for inclusion in the systematic review based on the preferred reporting items for systematic reviews and meta-analyses —extension for scoping reviews (PRISMA-ScR).

The initial search identified a total of 270 records. After removal of duplicates, titles and abstracts of 207 records were screened and 188 were excluded for not meeting our pre-defined inclusion criteria. Full texts for 19 reports were screened. Of these, 11 articles were excluded: *n* = 5 exposure didn't match inclusion criteria (reduced rank regression response variable didn't include dietary fat), *n* = 4 did not have a full-text available, *n* = 1 was a duplicate of another study and *n* = 1 had an ineligible study design (meta-analysis). In total, eight studies were deemed eligible and were included in the present review (Figure 1).

### Study characteristics

The description of the studies included in this scoping review is presented in Table 1. Six studies had a prospective design (16, 26, 28, 30, 32) and two were cross-sectional (27, 33). All studies included both male and female participants, except for one study, which investigated the odds of developing GDM in pregnant women (33). Sample sizes ranged from 249 (33) to 120,343 (31). Three studies were conducted in the United Kingdom (26, 28, 31) and others were conducted in Germany (16) Sweden (32) Australia (30) United States (33) and Malaysia (27). Most of the studies (*n* = 6) were conducted in adults (aged 18 to 69 years), except for two in adolescents (27, 30). One study was conducted in adults with T2D (16) and one in severely obese adults (32). All other studies (*n* = 6) were conducted in healthy populations (26–28, 30, 31, 33).

**Table 1:** Summary of studies investigating the association between *a posteriori* dietary patterns (reduced rank regression) and T2D and related outcomes [insulin resistance, HOMA-IR, fasting insulin, fasting glucose, oral glucose tolerance test (OGTT) and gestational diabetes].

**References, Country**	**Study design, population**	**Dietary assessment method**	**Dietary patterns**		**T2D outcomes**	**Adjustments**	**Results**
			**Predictors**	**Response variables**			
Pastorino et al. (26) United Kingdom	Prospective cohort, n at baseline = 1180, 55.5% female Age: 16-35y at baseline Follow-up: up to 11 years	Five-day food diary	45 food groups (g/day)	Dietary fiber density (g/1000kcal), Glycemic Index (units) Total dietary fat density (g/1000kcal)	Doctor diagnosed self-report T2D (yes/no; binary)	Demographic characteristics: Socioeconomic position, education Dietary intake: energy intake, energy misreporting Lifestyle risk factors: smoking, physical activity, medications for hypertension and dyslipidemia, BMI and waist circumference	An increasing trend in OR for T2D with increasing quintile of DP z-scores in women. Highest z-scores quintiles had: OR: 5.45, 95% CI: 2.01, 14.79
Hoffman et al. (16) Germany	Prospective cohort, n at baseline = 193 (cases of T2D) and 385 (controls) Each case matched by age and sex to 2 controls Follow-up: up to 3 years Age: Men 40 – 65y and women 35 – 64y at baseline	FFQ	49 food groups (g/day)	PUFA:SFA, dietary fiber (g/day), magnesium intake (g/day) alcohol (g/day)	Self-reported T2D development (yes/no; binary)	Demographic characteristics: age, sex, education Dietary intake: total energy intake Lifestyle factors: BMI, waist-hip ratio, sports activity, smoking status	DP was associated with lower risk of T2D. Relative risk from highest quintile: 0.49(95% CI: 0.25, 0.94)
Appannah et al. (30) Australia	Prospective cohort study, n at baseline = 1,605, 49% female Age at baseline: 14y Follow-up: up to 3 years	2 Semi-quantitative FFQ	47 food groups (g/day)	Dietary energy density (kj/g), fiber density (g/MJ), percentage of energy from total fat (%)	Serum Glucose (mmol/L; continuous), Insulin (%; continuous) and HOMA (%; continuous)	Demographic characteristics: age Dietary intake: energy misreporting Lifestyle factors: smoking status, physical fitness, BMI z-scores	A 1 SD unit increase in DP z-score was associated with higher fasting glucose in boys and higher insulin and HOMA in both boys and girls. Glucose: 0.04 (95% CI: 0.01, 0.08) Insulin: 3% (95% CI: 1%, 7%) HOMA: 4% (95% CI: 1%, 7%)
Brayner et al. (28) United Kingdom	Prospective cohort study, n at baseline = 16,523, 53.2% female Age: 40-69y at baseline Follow-up: mean 6.3 years	≥2 dietary assessment (Oxford WebQ)	48 food groups (g/day)	MUFA (%E), PUFA (%E), SFA (%E)	Doctor diagnosed self-reported T2D (yes/no; binary)	Demographic characteristics: age, sex, Townsend deprivation index Dietary intake: energy misreporting Lifestyle factors: physical activity, smoking status, BMI, blood pressure medication use and family history	The DPs were NS associated with T2D.
Gao et al. (31) United Kingdom	Prospective cohort study, n at baseline = 120,343, 56.5% female Age: 40-69y at baseline Follow-up: mean 8.4 years	≥2 dietary assessment (Oxford WebQ)	50 food groups (g/day)	Dietary energy density (kJ/g) SFA (%E) free sugars (%E) fiber density (g/MJ)	Diabetes incidence – from hospital admission registries (yes/no; binary)	Demographic characteristics: ethnicity, Townsend deprivation index, education Dietary intake: energy intake Lifestyle factors: smoking status, physical activity, family history (yes/no: diabetes, hypertension, cardiovascular disease, high cholesterol), menopause, BMI	A DP characterized by high intakes of butter, low-fiber bread, sugars and preserves, chocolate and confectionary, was associated with T2D. HR: 1.09, 95% CI: 1.06, 1.12. The DP characterized by high intake of sugar-sweetened beverages, fruit juice, table sugars and preserves and low intake of high-fat cheese and butter was NS associated with T2D.
Emi et al. (27) Malaysia	Cross-sectional study, *n =* 335, 68% female Age: 13y	FFQ	13 food groups (g/day)	Dietary energy density (kJ/g), fiber density (g/MJ), percentage of energy from total fat (%E) and from free sugars (%E)	Serum High HOMA (4.0 unit; binary), high serum insulin (25.0uIU/mL; binary), high blood glucose (5.6mmol/L; binary)	Demographic characteristics: sex, school location, mother's educational level Dietary intake: energy misreporting Lifestyle factors: physical activity, and BMI z-score	The identified DPs were NS associated with high HOMA, high serum insulin or high glucose.
Johns et al. (32) Sweden	Prospective cohort study (in severely obese individuals), n at baseline = 6,897 (for dietary patters creation), females *n =* 4,264 and *n =* 2,307 (for longitudinal analysis), females *n =* 1,447 Follow-up: mean 10 years	Semi quantitative FFQ	39 food groups (g/day)	Dietary energy density (kcal/g), fiber density (g/kcal), percentage of energy from SFA (%E)	Serum Blood glucose (mmol/L; continuous), Serum insulin (mmol/L; continuous)	Demographic characteristics: sex, age, education level Lifestyle factors: physical activity, smoking status, medications for blood pressure, lipid reducing or diabetes, baseline concentrations of the outcome (i.e. blood glucose or serum insulin)	The DP z-scores were NS with blood glucose, but were associated with higher serum insulin Insulin: beta coefficient: 1.22, *p < * 0.001
Shin et al. (33) United States	Cross-sectional study (in pregnant women), *n =* 249 Age: < 25y: 6%, 25-29y: 43.6%, 30–34y: 35.2%, ≥35y: 15.2% Age: 16-41y	24-hour recall	28 food groups (g/day)	Pregnancy BMI (kg/m^2^), dietary fiber (g/day), PUFA + MUFA: SFA	Gestational diabetes mellitus (yes/no; binary) defined by fasting plasma glucose level 5.1 mmol/L before 24 weeks of gestation	Demographic characteristics: age, race/ethnicity, family poverty income ratio, education, marital status Dietary intake: energy intake Lifestyle factors: physical activity, pregnancy BMI and gestational weight gain	Highest tertile of “high in refined grains, fats, oils and fruit juice” pattern had higher odds of GDM (OR: 4.9; 95% CI: 1.4, 17.0). Highest tertile of “high nuts, seeds, fat and soybean, low milk and cheese” pattern had higher odds of GDM (OR: 7.5; 95% CI: 1.8, 32.3). Highest tertile of “high added sugar and organ meats, low fruits, vegetables and sea foods” pattern had higher odds of GDM (OR: 21.1; 95% CI: 4.0, 109.8)

DP, dietary patterns; GDM, gestational diabetes mellitus; MUFA, monounsaturated fat; NS, not significant; PUFA, polyunsaturated fat; SFA, saturated fat; %E, percentage from total daily energy intake.

### Dietary intake assessments

Two studies used food frequency questionnaires (FFQ) to collect dietary data (16, 27) two used semi-quantitative FFQs (30, 32), two used a 24-hour dietary assessment tool (Oxford WebQ) (28, 31) one used 24-hour dietary recalls (28, 31, 33) and one used food diaries (Table 1) (26).

### Dietary patterns

In terms of dietary pattern predictor variables, food groups were presented in grams per day in all studies, with the number of food groups ranging from 13 (27) to 50 (Table 1) (31). All studies included a rationale for food groups creation. Detailed information on the included food groups is presented in Table 2. Regarding dietary pattern response variables, three studies included a variable for total fat intake: two as percentage from total energy intake (%E) intake (27, 30) and one as fat density (g/1000kcal) (26). Two studies included %E from SFA (29, 33) and one used %E from SFA, PUFA and MUFA (25). Two studies used a ratio of individual dietary fats: one used PUFA:SFA (16) and one used MUFA+PUFA:SFA (33). Seven of the eight included studies used other dietary components in addition to dietary fat as response variables. This included dietary fiber density (g/MJ) (26, 27, 30–32), fiber intake (g/day) (16, 33) glycemic index (%) (26) magnesium intake (g/day) (16) alcohol intake (g/day) (16) %E from free sugars (27, 31) with one study including an anthropometric component [Body Mass Index (BMI kg/m^2^)] (33). One study included only dietary fats as response variables (28). As summarized in Table 2, each study reported results for multiple dietary patterns. One study reported results for all dietary patterns generated (33). Four studies included dietary patterns that explained as much of the response variable as possible, using subjective cut offs in explained variation ranging from 10 to 20% (26, 28, 30, 31). Three studies only included one of the dietary patterns generated (16, 27, 32).

**Table 2:** Summary of food groups and dietary patterns of included studies.

**References**	**Food groups**	**Dietary patterns**
Pastorino et al. (26)	Food groups were created based on their response variables (glycaemic index, fat and fiber) content. Food groups: Pizza, pasta, rice, cereals and other, high-fiber cereals, low-fiber cereals, white bread, whole meal bread, crisp and other bread, biscuit, pastry, cakes, whole milk, skimmed milk, low-fat dairy desserts, full-fat yogurt, low-fat yogurt, full fat dairy dessert, cream, butter and animal fat, cheese, eggs, oils, plant fat solid, plant fat solid low fat, fish, red meat, offal, white meat, processed meat, vegetables, pulses, fruit, potatoes, fried potatoes, nuts and seeds, soups, dressing and sauces, jam and chutney, table sugar, honey and syrup, confectionery, savory snacks, alcoholic drinks, squashes and juices, pure fruit juice, soft drinks, coffee and tea	Three dietary patterns were generated, but only DP1 was further investigated as it explained >15% of the variation in the response variables. The dietary pattern was characterized by low intake of fruit, vegetables, low-fat yogurt, wholemeal bread, high-fiber cereals and high intakes of white bread, processed meat, fried potatoes, butter and animal fat and added sugar.
Hoffman et al. (16)	Food groups were created based on their culinary usage and nutrient content. Food groups: Cooked vegetables, cabbage family, legumes, cooked potatoes, mushrooms, sauce, poultry, meat except fish and poultry, animal fat except butter, dessert, cake, cookies, confectionary, ice cream, jam, honey, chocolate spread, canned fruit, fruit juice, tea, muesli, cornflakes, pasta, rice, pizza, vegetarian dishes, garlic, wholemeal bread, other bread, olive oil, fresh fruit, raw vegetables, other vegetable oils and fats, water, fish, nuts, chips, salt sticks, fried potatoes, beer, spirits, wine, other alcoholic beverages, eggs, coffee, soup, processed meat, low-fat dairy products, high-fat dairy products, low-fat cheese, high-fat cheese, butter, margarine	Although four dietary patterns were generated, food group correlations were only provided to dietary pattern 4, the only dietary pattern associated with type 2 diabetes. The dietary pattern was characterized by high intake of whole grain bread, fresh fruit, spirits and wine and low intake of low-fat and high-fat dairy, coffee, fruit juice, processed meats and margarine.
Appannah et al. (30)	Food groups creation was based on nutrient profiles or culinary usage, and their hypothesized contribution to diet–disease relationships Food groups: High-fat milk and cream, low-fat milk, yogurts, cheese, butter and animal fat, margarine and vegetable oils, eggs and egg dishes, low-fiber bread, high-fiber bread, other bread products, high-fiber breakfast cereals, other breakfast cereals, rice, pasta, and other grains, cereal-based mixed meals, pizza, biscuits and cakes, puddings, ice creams, chocolate and confectionery, sugar-free confectionery, spreads, meat and poultry, meat mixed dishes, processed meat, coated or breaded meat and fish, meat substitutes, fish, fried or roast potatoes, boiled or baked potatoes, vegetables (raw or boiled), fried vegetables, legumes, vegetable mixed dishes, fresh fruit, other fruit, nuts and seeds, crisps and savory snacks, soups, sauces (low energy dense), sauces (high energy dense), condiments, sugar-sweetened beverages, low-energy beverages, fruit juice, hot and powdered drinks, water, alcoholic drinks	Although this study generated three dietary patterns, only dietary pattern 1 was further investigated as it explained >15% of the variation in the response variables. The dietary pattern generated was characterized by high intakes of processed meat, chocolate and confectionery, low-fiber bread, crisps and savory snacks, fried and roasted potatoes and low intakes of fresh fruits, vegetables, legumes, high-fiber bread and yogurts
Brayner et al. (28)	Food groups were created based on the food groupings used in the UK National Diet and Nutrition Survey and were adapted according to differences in the response variables (SFA, PUFA, and MUFA) content of food items Food groups: Pasta, rice and cereals, whole meal pasta, rice and cereals, white bread, whole meal bread, high fiber breakfast cereals, other breakfast cereals, whole milk, skimmed milk, other milk, cheese, low fat cheese, yogurt low fat, yogurt full fat, ice cream, cream and dairy desserts, butter, margarine, olive oil, high-fat sauces, low-fat sauces, bacon and ham, beef and veal, non fried chicken, turkey pork and dishes, fried poultry, other meats, white fish, battered and fish products, oily fish, other seafood, eggs and eggs dishes, meat alternatives, vegetables raw and boiled, vegetables (mixed dishes), legumes, fruits, boiled and baked potato, soups, nuts and seeds, crisps, chips and savory snacks, buns, cakes, pastries and fruit pies, puddings, biscuits, sugar, preserves and confectionery, fruit juice, high sugar beverages, soft drinks, diet, tea and coffee, water, spirits and liqueurs, wine, beer and cider	Three dietary patterns identified, but only dietary patterns 1 and 2 were further investigated as they explained >10% of the variation in the response variables. Two dietary patterns were identified: one was characterized by higher intake of nuts, seeds, and butter and lower intake of fruit and low-fat yogurt and the other was characterized by higher intake of butter and high-fat cheese and lower intake of nuts and seeds.
Gao et al. (31)	Food groups were created aligned to the U.K. National Diet and Nutrition Survey and according to the similarity of their nutritional composition and culinary use. Food groups: Chocolate and confectionary, butter and other animal fat spreads, low-fiber bread, table sugars and preserves, grain-based desserts, sugar-sweetened beverages and other sugary drinks, high-fat cheese, crisps and savory snacks, alcoholic drinks (wine, beer, spirits), milk-based desserts, processed meat, red meat, high-fat milk and cream pizza, fried or roast potatoes, other bread products, coated or breaded meat and fish, fruit juice, egg and egg dishes, animal fat spread lower fat, plant-based spread normal, sauces and condiments, low/non sugar ssbs, nut-based spreads, milk-based and powdered drinks, sauces and condiments (low-fat), vegetable side dishes and dips, plant-based spread lower fat, pasta and rice, nuts and seeds, poultry, olive oil, other breakfast cereals, coffee and tea, other fish, low fat cheese, high-fiber bread, low-fat milk, oily fish, non-dairy milk, meat substitutes, legumes and pulses, boiled or baked potatoes, soups, wholemeal pasta and rice, dried and stewed fruit, high-fiber breakfast cereals, vegetables, fresh fruit.	Four dietary patterns were generated, but only 2 were further investigated as they explained >20% of the variation in the response variables. Two dietary patterns were identified: one was characterized by high intakes of chocolate and confectionery, butter, low-fiber bread, and sugars and preserves, and low intakes of fruits and vegetables and the other was characterized by high intakes of sugar-sweetened beverages, fruit juice, table sugars and preserves, and low intakes of high-fat cheese and butter.
Emi et al. (27)	Food groups were created based on their nutritional characteristics Food groups: Cereal and cereal based dishes, meat and poultry, seafood and shellfish, milk and dairy products, egg and egg dishes, nuts, vegetables, fruits, local desserts, sweet sweetened beverages, sweets, processed food, fast foods and snacks	Four dietary patterns were generated, but only dietary pattern 1 was further investigated as it explained the most variation in response variables (35%). The dietary pattern identified was characterized by high intakes of sugar-sweetened beverages, fruits, sweets and low intakes of meat and cereal
Johns et al. (32)	Food groups were created based on according to usual culinary usage and within the constraints of the food groupings in the dietary questionnaire Food groups: Chocolate, low fiber bread (swedish), full fat spread, cheese, fast food, cake, white bread, candy, fatty meat, full fat milk, pizza, crisps, soft drink, cookie, semi skimmed milk, nuts, oil, hot drink, dessert, spirits, beer, jam, potatoes, lean meat, egg, low fat spread, wine, skimmed milk, juice, full fat yogurt, light meals, fish, crisp bread, meat alternative, wholemeal bread, cereal, low fat yogurt, vegetables, fruit.	Three dietary patterns were generated, but only dietary pattern 1 was further investigated as it explained the majority of the variation in the response variables (54%). The dietary pattern identified was characterized by higher intake of chocolate, low-fiber bread, cheese, fast food, and cake and low intake of fruit and vegetables
Shin et al. (33)	Food groups were created based on grouping schemes reported in the Food Patterns Equivalents Database (FPED) 2011–2012 Food groups: Citrus, melons, and berries, other fruits, fruit juice, dark green vegetables, tomatoes, other red and orange vegetables (excludes, tomatoes), potatoes (white potatoes), other starchy vegetables (excludes white potatoes), other vegetables, beans and peas computed as vegetables, whole grains, refined grains, meat (beef, veal, pork, lamb, game), cured meat (frankfurters, sausage, corned beef, cured ham and luncheon meat made from beef, pork, poultry), organ meat (from beef, veal, pork, lamb, game, poultry), poultry (chicken, turkey, other fowl), seafood high in n-3 fatty acids, seafood low in n-3 fatty acids, eggs, soybean products (excludes calcium fortified soy milk and mature soybeans), nuts and seeds, milk (includes calcium fortified soy milk), yogurt, cheese, oils, solid fats, added sugars, alcoholic drinks	Three dietary patterns were identified: the first was characterized by high intake of refined grains, solid fats, oils, and fruit juice; the second was characterized by high intake of nuts and seeds, solid fats, soybean products and low loadings of milk and cheese; the third one was represented by high intake of added sugars and organ meats and low loadings of fruits and vegetables and seafood

### Type 2 diabetes and related outcomes

Four studies investigated T2D incidence (16, 26, 28, 31), two studies investigated associations with HOMA, fasting insulin and glucose levels (27, 30), one study investigated associations with fasting insulin and glucose levels (32) and one study investigated associations with GDM (Table 1) (33).

### Adjustments

All included studies adjusted for demographic characteristics, dietary intake and/or lifestyle characteristics (Table 1). All studies adjusted for sex, age, socioeconomic position, education or ethnicity (16, 26–28, 30–33). Seven studies adjusted for either total energy intake or energy intake misreporting (16, 26–28, 30, 31, 33). A combination of some lifestyle factors such as physical activity, BMI, smoking, family history of T2D and/or hypertension and waist to hip ratio were adjusted for in all studies (16, 26–28, 30–33).

### Dietary patterns and type 2 diabetes

Four of the eight studies were prospective studies examining T2D incidence (Table 1). Of these, two studies identified a high-saturated fat dietary pattern associated with higher risk of T2D (26). These studies (*n* = 2) reported lower intake of foods such as fruit and vegetables (Table 2). Pastorino et al. (26) identified a high-fat, high-GI, low-fiber dietary pattern, characterized by high intake of butter, animal fat and processed meat and low intake of fruits and vegetables, that was associated with higher odds of developing T2D in women (OR: 5.45; 95% CI: 2.01 to 14.79), but not in men. Hoffman et al. (16) generated a low PUFA:SFA ratio and magnesium and high alcohol and fiber pattern, which was significantly associated with lower risk of T2D (relative risk of highest vs lowest quintile: 0.49, 95% CI: 0.25, 0.94). Brayner et al. (28) aimed to investigate the prospective associations between fat-derived dietary patterns and obesity, abdominal obesity and T2D incidence. Of the two dietary patterns investigated, a high SFA and low PUFA and MUFA dietary pattern was associated with higher obesity and abdominal obesity (OR: 1.24, 95% CI: 1.02, 1.45; and OR: 1.19, 95% CI: 1.02, 1.38, respectively), but not with T2D. Gao et al. (31) investigated two dietary patterns. Of these a dietary pattern associated with high intake of chocolate, confectionary, butter, low-fiber bread and low intake of fruits and vegetables (high energy density, SFA, and free sugars and low fiber density pattern) was associated with higher risk of T2D (HR: 1.09, 95% CI: 1.06, 1.12).

### Dietary patterns and type 2 diabetes-related outcomes

Three of the eight studies investigated either prospective (*n* = 2) or cross-sectional (*n* = 1) associations with either HOMA, fasting insulin and/or glucose (Table 1). Of these, two studies identified a high-saturated fat dietary pattern associated with higher levels of T2D biomarkers such as fasting insulin, glucose and HOMA (30, 32). These studies reported lower intake of fiber-rich foods such as fruit and vegetables (Table 2). Appannah et al. (30) identified that an energy dense, high-fat, low fiber dietary pattern was associated with 0.04 mmol/L (95% CI: 0.01, 0.08) higher fasting glucose in boys, 3% (95% CI: 1%, 7%) higher insulin and 4% (95% CI: 1%, 7%) higher HOMA in both boys and girls. In contrast, Emi et al. (27) determined that a dietary pattern high in sugar, fiber, high dietary energy density and low fat, and characterized by high intake of sugar sweetened beverages, sweets and low intake of cereal and meat, was not associated with any T2D markers. Johns et al. (32) identified that an energy-dense, low-fiber and higher saturated fat dietary pattern characterized by higher intake of foods such as chocolate, low-fiber bread, cheese and fast food was not associated with blood glucose, but was associated with higher insulin levels (beta coefficient: 1.22 ± 0.17, *p* < 0.001). Lastly, Shin et al. (33) identified three dietary patterns, “high refined grains, fats, oils and fruit juice”, “high nuts, seeds, fat and soybean; low milk and cheese”, and “high added sugar and organ meats; low fruits, vegetables and seafood”, which were associated with higher odds of GDM (OR:4.9, 95% CI: 1.4, 17.3; OR: 8.2, 95% CI: 1.8, 37.4; OR: 21.1, 95% CI: 4.0, 109.8, respectively).

## Discussion

This is the first scoping review to synthesize information on the associations between dietary fats and risk of T2D in an *a posteriori* dietary patterns context. The main findings are that of the eight articles included, five high-fat dietary patterns were identified that were positively associated with either T2D incidence or elevated fasting glucose, insulin and/or HOMA levels. All five dietary patterns were characterized by other nutrients in addition to dietary fats, such as low dietary fiber and high dietary energy density, characterized by low intake of fruit and vegetables, reduced fat dairy products and higher intake of processed meats and butter. The only dietary pattern that used exclusively dietary fat as the response variables (namely PUFA, MUFA and SFA) did not find any associations with T2D risk. This suggests that intake of unhealthy fats is often accompanied by lower intake of fruits, vegetables and other fiber-rich foods, which may need to be considered when deriving *a posteriori* dietary patterns for assessing T2D risk. Therefore, consumption of healthy dietary fats for the prevention of T2D should be encouraged as part of a healthful overall dietary pattern.

Although all studies in this review used dietary fat as one of the response variables, the way these were used varied between studies. Regardless of whether total fat, or individual dietary fats were used, most dietary patterns generated included high factor loadings for meat and dairy (16, 26, 28, 30, 33). This is consistent with literature on the main food sources of saturated fat, where high processed meats intake has been consistently associated with higher risk of T2D and cardiovascular diseases (34, 35) whereas findings for dairy are mixed, and often depend on whether full fat or low fat dairy products are considered separately (36). For the two studies that included saturated and unsaturated fats as response variables, high factor loadings for nuts and seeds were also observed. However, findings from these studies were conflicting; Shin et al. (33) reported that a dietary pattern high in nuts, seeds, fat and soybean was associated with higher odds of developing GDM, whilst Brayner et al. (28) reported that a dietary pattern high in nuts, seeds and butter was not associated with T2D incidence. This difference may be partially attributable to the sample populations, since Shin et al. (33) examined GDM in pregnant women and Brayner et al. (28) examined T2D incidence in healthy adults. Taken together, these findings suggest that the underlying foods that contribute to a high-fat dietary pattern should be taken into consideration to ensure these foods are lower in SFA.

Outcomes from this review align with literature on the effect of dietary fats on fasting serum glucose and insulin levels. The study by Appannah et al. (30) reported an association between a dietary pattern characterized by high intakes of high-SFA foods, such as processed meat, chocolate, savory snacks and fried foods, and higher fasting serum glucose and insulin, which could indicate insulin resistance. This supports evidence from randomized controlled trials (RCT), which have shown that high-SFA diets can decrease insulin sensitivity (37, 38). For example, a 12-week RCT of 486 European adults investigated two high-fat (either high in SFA or high in MUFA) and two low-fat (with or without 1.2 g/day n-3 PUFA supplementation) isocaloric diets. In healthy individuals, the high MUFA diet increased participants' insulin response to glucose, whilst the high SFA diet reduced insulin response (37). Similarly, a meta-analysis of RCTs reported that replacing 5% of energy from SFA with PUFA lead to significant decreases in glucose, HbA1c and HOMA levels. Although the mechanisms behind high-SFA diets and insulin resistance are still unclear, there is evidence to suggest that it might be linked to ceramide production (39). High-SFA diets increase ceramide production, which in turn can impair insulin signaling (39). High-SFA diets have also been suggested to be more inflammatory and chronic low-grade inflammation can inhibit insulin action (40, 41). Although RCTs can provide insight into the causal relationship between dietary fats and glucose or insulin, prospective cohort studies can better understand these associations in a dietary pattern context. Moreover, as fasting glucose and insulin are important biomarkers in T2D development, more prospective studies investigating their long-term associations are needed to better elucidate the associations between dietary fat and T2D incidence in an *a posteriori* dietary pattern context.

Consistent with this review, evidence suggests that intake of specific nutrients, in addition to dietary fats, may play an important role in the development of T2D (42). Higher dietary fiber, found in fruits and vegetables, for instance, has been shown to modulate blood glucose concentration following a meal (43) and has thus been linked to a lower risk of T2D (5). Conversely, higher intake of carbohydrate, especially refined carbohydrate has a strong positive association with T2D risk (5) High dietary sodium is also linked to insulin resistance (44). Dietary patterns such as Dietary Approaches to Stop Hypertension (DASH) and the Mediterranean diet have dietary fat as a key focus, but are also a rich source of other key nutrients, such as fiber and antioxidants (19). A systematic review and meta-analysis of 48 studies has shown that a higher adherence to either DASH (RR: 0.81; 95% CI: 0.72, 0.92) or Mediterranean diet (RR: 0.87; 95% CI: 0.82, 0.93) was associated with substantial reduction in T2D incidence. Both dietary patterns encourage a high consumption of fiber-rich foods such as fruits and vegetables, along with lower intakes of SFA rich foods such as red meat and processed foods (45, 46). Therefore, the combination of dietary fats with other dietary components may be a stronger predictor for T2D, than any nutrient in isolation. However, as only one study was identified that derived dietary patterns based on dietary fat alone, further prospective evidence is needed to confirm this.

The present review provides supporting evidence for the role of energy-dense dietary patterns as a risk factor for T2D development (47). Half of the studies included in this review included energy density as a response variable (27, 30–32) which has an established positive association with obesity risk (48) whereas associations with T2D are less clear (49). Energy dense foods are also often high in SFA and added sugars, low in fiber and have a high glycaemic load, all of which have been linked to a higher risk of T2D (50). Interestingly, the study by Brayner et al. (28) in which only dietary fats were used to derive dietary patterns, there was no association with T2D. However, there was an association between higher SFA-rich foods and lower PUFA-rich foods with increased odds of developing overall and abdominal obesity. Therefore, although there are no clear associations between dietary fats and T2D, it may be that obesity is on the causal pathway as a key risk factor for T2D development (51). As many as 85% of individuals with T2D have either overweight or obesity (52). Further, obesity can contribute to low-grade chronic inflammation (53) whereas PUFA intake can counteract pro-inflammatory pathways (54).

This review has identified some discrepancies in the design and reporting of reduced rank regression methods that limit the interpretability of results. As the number of dietary patterns generated depended on the number of response variables, studies included in this review ranged from three to four response variables (16). This created challenges when the number of patterns reported differed, as the rationale for which dietary patterns to report also varied. Often, the dietary patterns that explained the most variation in the response variables were reported, regardless of how much was explained by the other dietary patterns. However, this cut off ranged from >10% to 20% (26, 28, 30, 31) of explained variation, with some studies not providing a numerical cut off (16, 27, 32, 33), The number of food groups also varied, ranging from 13 to 50. Evidence from principal component analysis, another data-driven dietary pattern method, has shown that changes in the number of food groups can influence the associations with health outcomes (16). Thus, more consistent definition of this reporting of minimum cut off points and the rationale for classifying food groups would improve the reporting of this dietary pattern method in the literature.

This review acknowledges some strengths and limitations. Firstly, as a scoping review, this study followed systematic searching methods based on PRIMSA-ScR guidelines. Secondly, it identified reporting discrepancies in the use of reduced rank regression that should be addressed. In line with scoping review methodology, no critical appraisal of the evidence has been conducted, and therefore the quality of studies has not been considered. However, most studies were large prospective studies that adjusted for demographic characteristics, dietary intake and lifestyle risk factors.

In conclusion, this scoping review has identified eight studies using reduced rank regression to derive dietary patterns based on dietary fat. Overall, findings suggest that high-fat dietary patterns, especially high-SFA containing foods, were positively associated with T2D incidence and glucose, insulin and HOMA levels. While dietary fat may be an important predictor of T2D risk, evidence from a dietary pattern perspective suggests that foods such as processed meats, low-fiber cereals and low intakes of fruit and vegetables are likely to contribute to an increased T2D risk. Therefore, consumption of healthy dietary fats for the prevention of T2D should be encouraged as part of a healthful overall dietary pattern.

## Data availability statement

The original contributions presented in the study are included in the article/Supplementary material, further inquiries can be directed to the corresponding author.

## Author contributions

BB and LM performed the screening of articles. BB drafted the manuscript. KL reviewed first draft of the manuscript. All authors contributed to the design of the study, the interpretation and critical evaluation of the review, provided edits, and approved final submission of the manuscript.
