# Supplementary material for: Novel approach to investigate the association between type 2 diabetes risk and dietary fats in a dietary pattern context: a scoping review
Source: Front Nutr. 2023 Jun 1;10:1071855. doi: 10.3389/fnut.2023.1071855 (PMC10267339; doi:10.3389/fnut.2023.1071855)
Supplement: Supplementary file 1 [file Table_1.DOCX]

Supplementary Material

**Supplementary table**

**MEDLINE complete search strategy**

| 1 | (“reduced rank regression” or “rrr”) and (“type 2 diabetes” or “T2D” or “T2DM” or “type 2 diabetes mellitus” or “TIIDM” or “insulin resistance” or “insulin resistant” or “IR” or “impaired glucose tolerance” or “pre-diabetes” or “glucose intolerance” or “impaired fasting glucose” or “gestational diabetes” or “GDM”) |
| --- | --- |
| 2 | Using “English language only” filter |
